# Use of Nanoparticles in Tissue Engineering and Regenerative Medicine

**DOI:** 10.3389/fbioe.2019.00113

**Published:** 2019-05-24

**Authors:** Milad Fathi-Achachelouei, Helena Knopf-Marques, Cristiane Evelise Ribeiro da Silva, Julien Barthès, Erhan Bat, Aysen Tezcaner, Nihal Engin Vrana

**Affiliations:** ^1^Department of Biomedical Engineering, Middle East Technical University, Ankara, Turkey; ^2^Inserm UMR 1121, 11 rue Humann, Strasbourg, France; ^3^Protip Medical, 8 Place de l'Hôpital, Strasbourg, France; ^4^Department of Tests in Materials and Products, Instituto National de Tecnologia, Rio de Janeiro, Brazil; ^5^Department of Chemical Engineering, Middle East Technical University, Ankara, Turkey; ^6^Department of Biotechnology, Middle East Technical University, Ankara, Turkey; ^7^Department of Engineering Sciences, Middle East Technical University, Ankara, Turkey; ^8^BIOMATEN, METU, Center of Excellence in Biomaterials and Tissue Engineering, Ankara, Turkey

**Keywords:** tissue engineering, regenerative medicine, metallic nanoparticles, ceramic nanoparticles, polymeric nanoparticles, nanoparticles in bioinks

## Abstract

Advances in nanoparticle (NP) production and demand for control over nanoscale systems have had significant impact on tissue engineering and regenerative medicine (TERM). NPs with low toxicity, contrasting agent properties, tailorable characteristics, targeted/stimuli-response delivery potential, and precise control over behavior (via external stimuli such as magnetic fields) have made it possible their use for improving engineered tissues and overcoming obstacles in TERM. Functional tissue and organ replacements require a high degree of spatial and temporal control over the biological events and also their real-time monitoring. Presentation and local delivery of bioactive (growth factors, chemokines, inhibitors, cytokines, genes etc.) and contrast agents in a controlled manner are important implements to exert control over and monitor the engineered tissues. This need resulted in utilization of NP based systems in tissue engineering scaffolds for delivery of multiple growth factors, for providing contrast for imaging and also for controlling properties of the scaffolds. Depending on the application, materials, as polymers, metals, ceramics and their different composites can be utilized for production of NPs. In this review, we will cover the use of NP systems in TERM and also provide an outlook for future potential use of such systems.

## Introduction

Due to many drawbacks of tissue and organ transplantation such as limited donor availability, the need for immunosuppression and insufficient success rate (rejection of the transplant), there is an increasing demand in tissue engineering and regenerative medicine (TERM) solutions which is a rapidly growing multidisciplinary field. It has merged the biological, material and engineering sciences to develop and manufacture artificial structures that resemble the native tissue/organ not only as implantable systems but also as model, miniaturized organs (Dvir et al., 2011; Zorlutuna et al., 2013). Mimicking the natural extracellular matrix (ECM) composition of a tissue through constructing a three dimensional (3D) scaffold for cells with appropriate mechanical strength, ease of monitoring cellular activities and delivering of bioactive agents require a nanoscale approach rather than a macroscopic one to obtain satisfactory results. Nanoparticles (NPs) can provide high control over properties of scaffolds such as tuning their mechanical strength and providing controlled release of bioactive agents (Park et al., 2012; Pérez et al., 2013; Cheng et al., 2015; Bahal et al., 2016; Mi et al., 2016). Additionally, drawbacks and limiting factors such as low solubility, unstable bioactivity and short circulation half-life of bioactive molecules (growth factors, cytokines, inhibitors, genes, drugs etc.) and contrast agents have made the NPs as one of the most suitable candidates for bioactive agent delivery and monitoring for applications (Park et al., 2012; Pérez et al., 2013; Cheng et al., 2015; Bahal et al., 2016; Mi et al., 2016).

**Figure 1:**
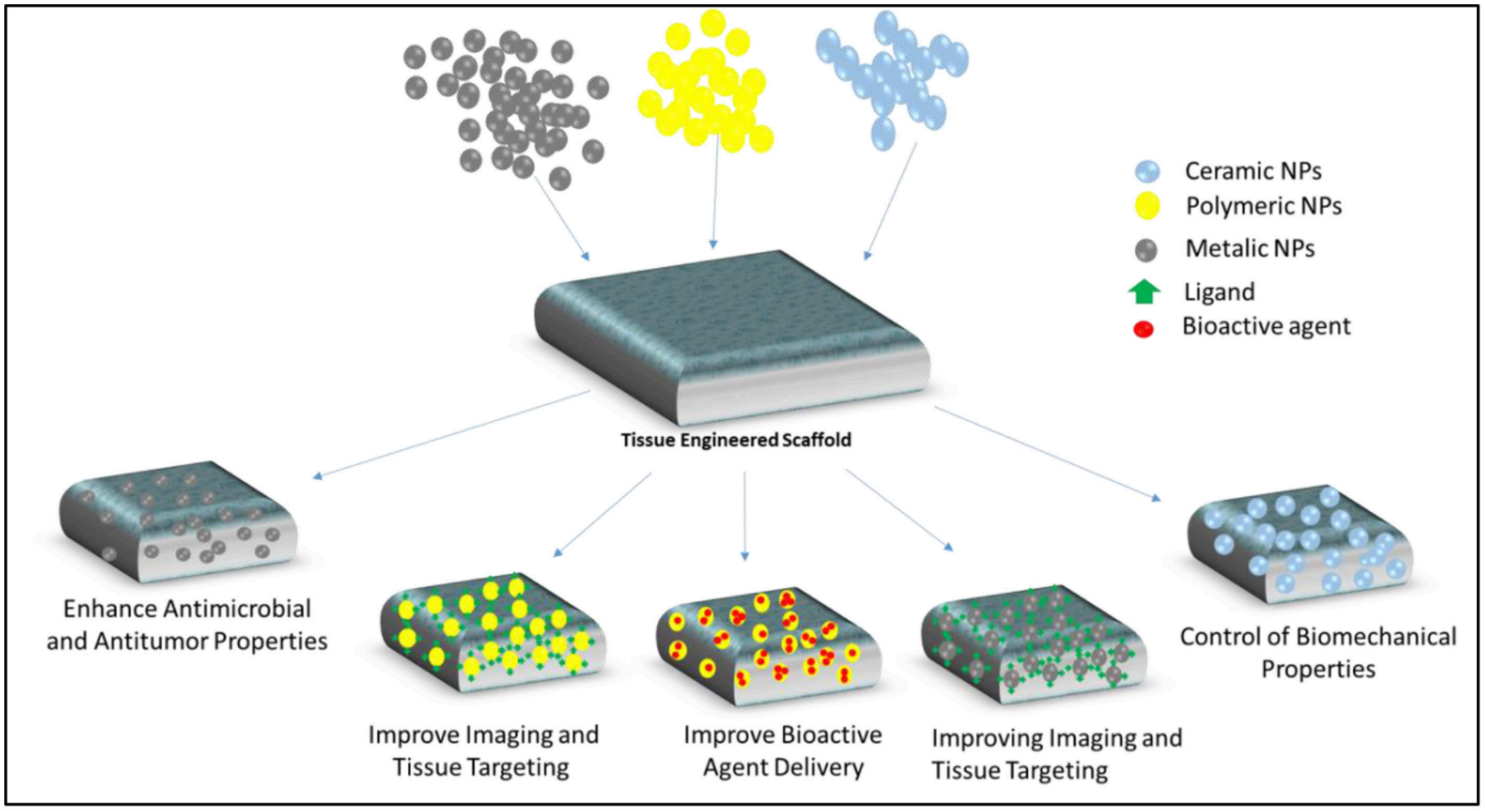
Examples of different types (ceramic, polymeric, metallic) nanoparticles which can be utilized for various applications in TERM including tissue targeting and imaging, bioactive agent delivery, modulating mechanical properties of scaffolds, providing antimicrobial and antitumor properties.

Nanotechnology as a processing technology includes synthesizing NPs and using them for a wide range of applications. NPs with sizes ranging from ~ 10 to 1,000 nm can be prepared in solid and colloidal forms (Colson and Grinstaff, 2012). NPs have vast area of applications in the production of sensors, photovoltaic devices, and biomedical field such as drugs delivery and vaccine adjuvants (Saroja et al., 2011; Meng et al., 2012; Saha et al., 2012; Stratakis and Kymakis, 2013; Shang et al., 2014). The impact of nanotechnology has altered traditional and simple approaches in TERM toward more complex and efficient systems. Along NPs, other products of nanoscale technology such as nanofibers and nanopatterned surfaces have been used for directing cell behavior in TERM field. Utilizing simultaneous therapeutic and imaging systems, embedding novel biomaterials with superior spatiotemporal control within scaffolds, modulating release of multiple bioactive agents especially growth factors to direct fate of stem cells and morphogenesis, adjusting mechanical strength of scaffolds for hard tissue applications, and minimizing toxicity and increasing biocompatibility through tissue specific delivery are among various applications of NPs in TERM (Figure 1) (Shi et al., 2010; Colson and Grinstaff, 2012).

NPs can be prepared with various types of materials such as ceramics, metals, natural and synthetic polymers. Their compositions and characteristic advantages like high penetration ability, high surface area with tunable surface properties make them as one of the widely preferred candidates in TERM field for imaging, mechanical strength enhancement, as bioink supplements, antimicrobial, and bioactive agent carriers (Figure 1) (Shi et al., 2010; Colson and Grinstaff, 2012).

In this article, metallic, ceramic, and polymeric NPs with an emphasis on their TERM applications are reviewed.

## Metallic Nanoparticles

NPs provide a link between bulk materials and molecular or atomic structures (Salata, 2004). Metallic NPs can be manufactured and modified through utilizing different functional groups that provide conjugation of antibodies, ligands, and drugs as delivery systems (Dobson, 2006; Mody et al., 2010). This section summarizes some examples of metallic NPs with respect to biomedical applicability concerning gold and silver NPs.

### Gold Nanoparticles

Gold nanoparticles (AuNPs) can be described as a colloid of nanometer sized particles of gold. Colloidal gold solutions present different properties compared to the bulk gold, for example, their optical property due to their unique interaction with light (Daniel and Astruc, 2004). Turkevich et al. synthesized monodisperse spherical gold NPs for the first time (Turkevich et al., 1951). This method was then modified by others (Frens, 1973; Kimling et al., 2006). On gold surface it is possible to conjugate various ligands including polypeptide sequences, antibodies and proteins with various moieties such as phosphines, amines, and thiols, as their strong affinity to gold is known (Alivisatos et al., 1996).

**Figure 2:**
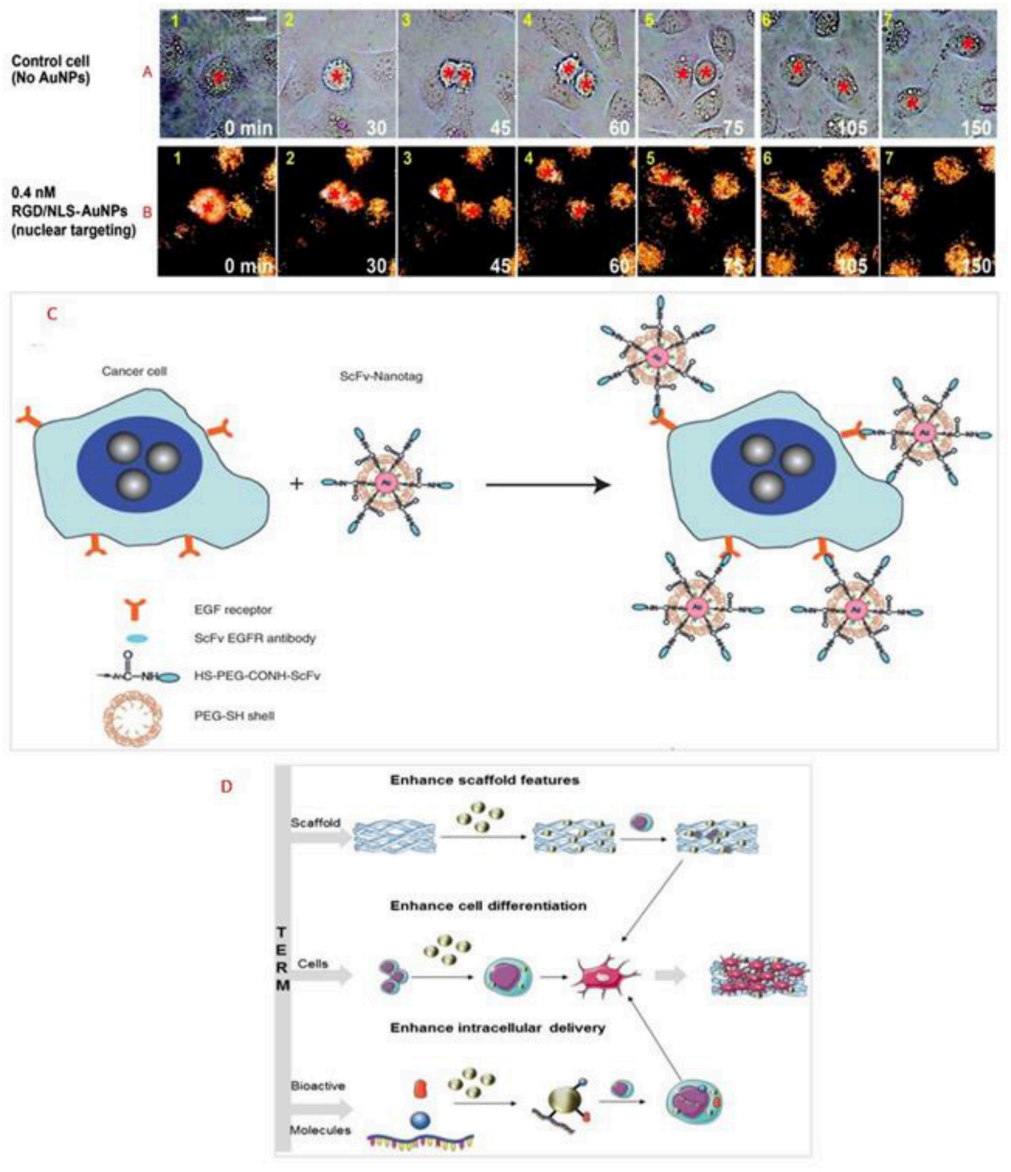
Real-time images of cancer cell division under the following conditions: **(A)** with No AuNPs and **(B)** in the presence of 0.4 nM nuclear-targeting gold nanoparticles (RGD/NLS-AuNPs). Red stars indicate the nuclei. Scale bar: 10 μm. Reprinted from Kang et al. (2010) with permission from American Chemical Society. **(C)** Preparation of targeted surface-enhanced Raman scattering (SERS) NPs by using a mixture of SH-PEG and a hetero-functional PEG (SH-PEG-COOH). Covalent conjugation of an EGFR-antibody fragment occurs at the exposed terminal of the hetero-functional PEG. Reprinted from Qian et al. (2008) with permission from John Wiley and Sons. **(D)** Scheme representing the use of AuNPs in tissue engineering and regenerative medicine. Reprinted from Vial et al. (2017) with permission from Elsevier.

One potential use of gold NPs in the context of regenerative medicine is as a safety measure if the implanted tissue is replacing a resected tissue/organ due to tumor growth. One example is the use of AuNPs for disturbing the cancer cell division by selectively transporting the particles into affected cells' nuclei. Kang and colleagues developed polyethylene glycol (PEG) coated AuNPs (30_nm) through binding it with nuclear localization signal (NLS) peptides together with arginine—glycine—aspartic acid (RGD) (Kang et al., 2010). Human oral squamous cell carcinoma (HSC) overexpressing αvβ6 integrins and human keratinocytes (HaCat) were utilized a cancer cells and normal cells, respectively, in this study. Figures 2A,B shows real-time monitoring of cancer cells in the absence (control) and with 0.4 nM RGD/NLS-AuNPs. For the first case, cytokinesis of control cells started at 45 min (Figure 2A3). Cytoplasmic bridge connected the daughter cells and this connection was extended over time (Figure 2A6). Total separation of two daughter cells was observed after 2 h (Figure 2A7). Nevertheless, complete cell division was not observed for cells that were incubated with 0.4 nM RGD/NLS-AuNPs. Cytokinesis continued similar to the control (Figures 2B1–4). In contrast to control group, cytoplasmic bridge did not extend after fully contraction of cleavage furrow (Figures 2B5–6) and consequently, daughter cells formed a binucleated cell (Figure 2B7). It was concluded that cytokinesis arrest (blockage of the final step in cell division) resulted through nuclear targeting of AuNPs in cancer cells therefore preventing cells from completing cell division (Kang et al., 2010). Nuclear targeting of the cancer cells plays a crucial role in the success of the cancer treatment. Recently, it was reported that *in situ* aggregation of non near-infrared (NIR) absorbing plasmonic AuNPs took place at the nuclear region of the cells (Panikkanvalappil et al., 2017) which makes plasmonic AuNPs as a suitable candidate for NIR photoabsorber for plasmonic based photothermal therapy in cancer. By shifting significantly the absorption band to NIR range, plasmonic AuNPs they protect healthy tissue through reducing heat-induced collateral damage. In another study, it has been shown that AuNPs targeting the cell nucleus membrane has increased the overexpression of laminin A/C and mechanical stiffness of nucleus and consequently decreased the cancer cell migration (Ali et al., 2017). All these properties of AuNPs can be utilized for targeting the remaining cancer cells following tumor resection and consequently minimizing cancerous cells remaining in the healthy tissue microenvironment. Therefore, applying AuNPs prior to implantation can provide a safety measurement toolbox to minimize the recurrence of tumor through targeted delivery to cancer cells, and consequently; increase the chance of the effective implantation for various TERM applications.

Moreover, AuNPs are widely used for drug delivery applications (Manivasagan et al., 2016; Amoli-Diva et al., 2017; Labala et al., 2017). They are also used as a probe for Raman scattering aimed for *in vivo* cell targeting. AuNPs can be conjugated to epidermal growth factor receptor (EGFR) through an antibody, for targeting tumor cells. The antibody fragment recognizes EGFR on cancer cells (Paez et al., 2004). After systemic administration, those AuNPs, were capable of intensifying the Raman scattering efficiency of adsorbed molecules nearly to 10^15^ times (Paciotti et al., 2006). Process of cell targeting is shown in Figure 2C. Qian et al. has prepared thiol-modified PEG coated AuNPs and compared it with AuNPs to understand their targeting efficiency using single-chain variable fragment antibodies (ScFv) as EGFR, and His-tagged green fluorescent protein (GFP) (Qian et al., 2008). PEG-coated, ScFv, and GFP bound NPs were able to directly aim biomarkers on the surface of tumor cells which were encoded with surface-enhanced reporter with a Raman reporter. Through utilization of surface-enhanced Raman scattering (SERS), recognition of human cancer cells with minimum passive aggregation and remarkably specific detection in xenograft tumors was reported by utilization of AuNPs coated with thiol-modified PEG. In the context of tissue engineering such systems can contribute to precise monitoring of potential relapse during the integration of the implanted system.

As reviewed by Vieira and colleagues, different NPs can be used for bone tissue engineering with emphasis on scaffolds' improvement and drug delivery (Vieira et al., 2017). Among NPs (organic and inorganic), AuNPs have been used in scaffolds for enhancing bone regeneration, due to their potential to promote cell differentiation (Zhang et al., 2014; Ko et al., 2015). Heo et al. presented an enhanced bone regeneration by using a complex composed of AuNPs and gelatin scaffolds (Heo et al., 2014). This combination leads to *in vitro* and *in vivo* osteogenic differentiation of adipose-derived stem cells. 2,2,6,6-Tetramethylpiperidine-N-oxyl (TEMPO) conjugated AuNPs have been reported to be efficiently uptaken by human mesenchymal stem cells (MSCs) and reduce the overproduction of reactive oxygen species in them at low dosage of TEMPO (Li J. et al., 2017). It has also enhanced osteogenic differentiation of human MSCs while suppressing the adipogenic differentiation. Consequently, it can be used for ROS-induced dysfunctions while regulating the desired differentiation type. Similarly, osteogenic differentiation of MSCs in fibrin and poly(caprolactone) (PCL)-based scaffolds containing PEGylated hollow AuNPs through increasing Runx2 gene expression has been reported (del Mar Encabo-Berzosa et al., 2017). RGD-modified AuNPs have been shown to have an effect in a ligand density dependent manner over the MSCs differentiation characteristics (Li J. et al., 2018). While high density of RGD resulted in decreased alkaline phosphatase (ALP) activity and enhanced the adipogenic marker gene expression, low density RGD caused a decrease in the oil droplet formation and adipogenic marker gene expression.

Vial and colleagues described different ways of how TERM can be affected by the use of AuNPs, as seen in Figure 2D (Vial et al., 2017). TERM combines the following elements: scaffold, cells, and bioactive molecules. The main objective of adding AuNPs is not only to improve scaffold structures, but also to guide cell behavior, which means enhancing cell differentiation and the intracellular delivery (Vial et al., 2017). This interaction between AuNPs and various cells could be in different aspects such as modulation of cardiomyocytes using calcium oscillation by heating AuNPs (via 532 nm picosecond pulsed laser) (Gentemann et al., 2017); stimulation of striated muscle cells with NIR using gold nanoshells as effective wireless stimulation technique for muscle tissue engineering through myotube activation (Marino et al., 2017); increase in migration of pro-healing M2 macrophages and proliferation of neonatal cardiomyocytes under electrical stimulation in collagen-silver/gold NPs 3D matrix (Hosoyama et al., 2017); and enhancement in dopaminergic neural differentiation of mouse embryonic stem cells (ESCs) through mTOR/p70S6K pathway using AuNPs of around 30 nm in size (Wei et al., 2017).

Chen et al. have reported the impact of the AuNPs on MSCs for vascular tissue engineering (Chen Y.-W. et al., 2018). Incorporation of 43.5 ppm AuNPs into fibronectin (FN) coat has decreased the elasticity of the coating of the composite material and enhanced its thermal stability. Hydrophilicity of the composite has increased compared to control groups, which would help the attachment of MSCs. FN loaded AuNPs which was coated over catheter has stable and slow degradation *in vivo*. MSCs treated with VEGF (vascular endothelial growth factor) (50 ng/ml) enhanced cell migration on the scaffold of FN-AuNPs via signaling pathway of matrix metalloproteinase (MMP)/ endothelial nitric oxide synthase (eNOS). MSCs have shown higher antithrombotic activity, better endothelialization and higher expression of CD31 and alpha-smooth muscle actin (α-SMA) with FN-AuNPs coated catheters implanted compared to control groups.

AuNPs also can be applied for wound healing applications. Akturk et al. have observed better wound closure in AuNPs containing wet electrospun silk fibroin compared to control groups (Akturk et al., 2016). Higher neovascularization and granulation tissue formation have also been observed compared to untreated skin control group.

### Silver Nanoparticles

**Figure 3:**
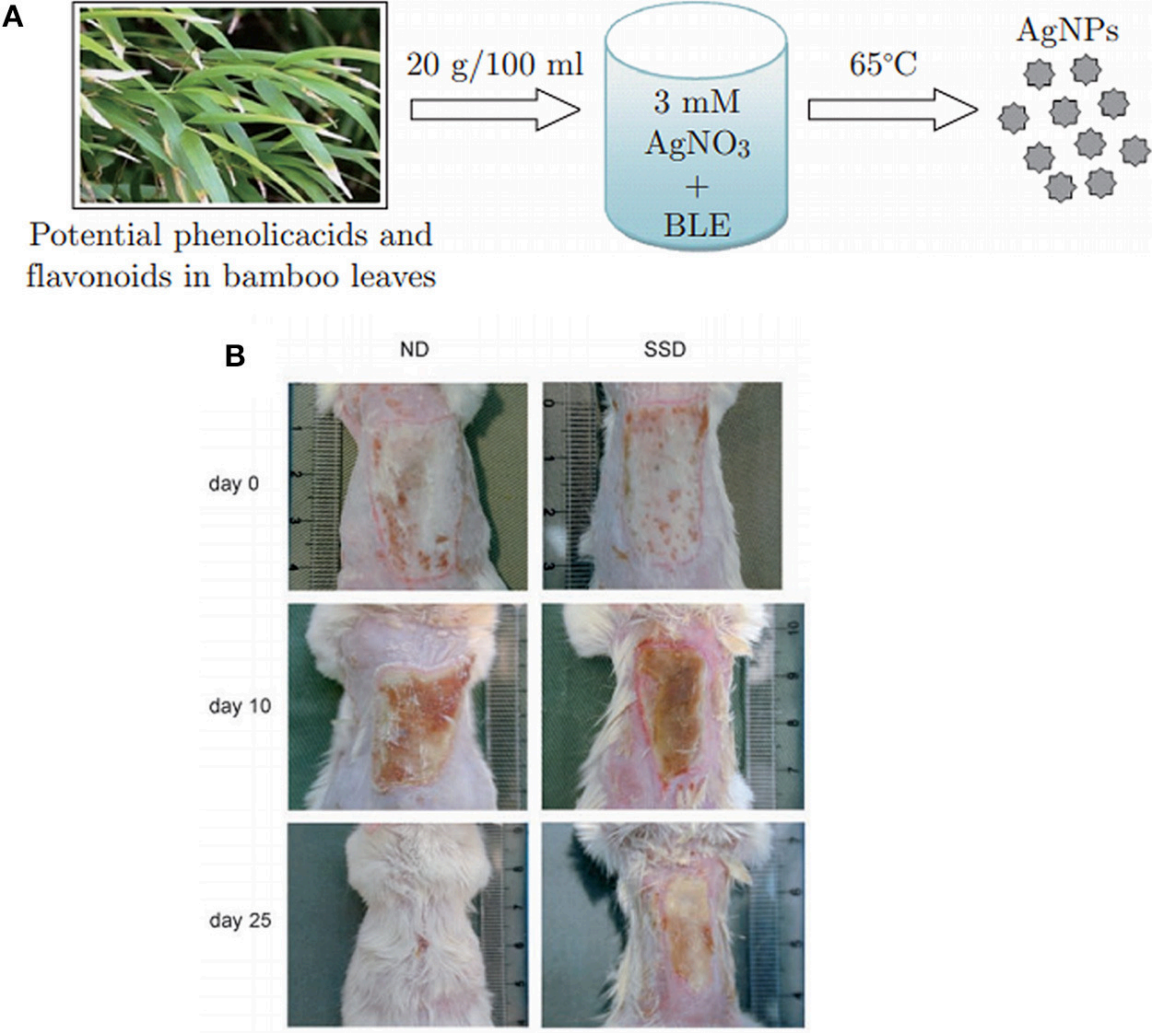
Effect of silver nanoparticles on burn wound healing. **(A)** Synthesis mechanism of silver nanoparticles using bamboo leaves extract (BLE) to reduce silver nitrate. Reprinted with permission from Yasin et al. (2013) **(B)** Images of burn wound from mouse treated with silver nanoparticles (ND) and silver sulfadiazine (SSD) at different time point of wound healing. Reprinted from Tian et al. (2007) with permission from Springer.

Silver nanoparticles (AgNPs) can also be described as a colloid of nanometer sized particles of silver and are one the most widely used metallic NPs in biomedical field mainly for their antimicrobial properties (Rai et al., 2009; Prabhu and Poulose, 2012). As bacterial infection is a significant risk with engineered tissues; use of AgNPs as a safety measure is a potential solution. These NPs can be produced by either physical or chemical processes (Panácek et al., 2006; Guzmán et al., 2009). The physical methods used to synthetize AgNPs are evaporation-condensation process, laser ablation of metallic bulk material, gamma irradiation or ultrasonic irradiation. Chemical methods are mostly based on the utilization of sodium borohydride or polyol as reducing agents in order to reduce silver salt solution (silver nitrate) (Frattini et al., 2005). After reduction of silver ions (Ag^+^) into metallic silver (Ag^0^), NPs will be formed through nucleation followed by the growth. In the past few years, biological methods using microorganisms such as bacteria or, eukaryotic fungi or plants to reduce silver ions, have emerged to synthetize AgNPs (Figure 3A). This method called bioreduction of silver ions is considered more eco-friendly since it does not involve the use of toxic chemicals during the process (Yasin et al., 2013; Ahmed et al., 2016). Depending on the method used (chemical or physical) and the choice of the reducing agent (weak or strong), the size of the NPs can range from few nanometers to more than 500 nm diameters. To stabilize and control the size of the particles, almost all methods comprise the use of surfactants (Iravani et al., 2014). Silver ions have been used for a long time for their antimicrobial properties toward a wide range of microorganisms. It has been shown that silver ions are able to block the microbial respiratory chain system and precipitate bacterial cellular protein (Abbasi et al., 2016). Morones et al. have studied the antimicrobial properties of AgNPs against four types of gram-negative bacteria (Morones et al., 2005). In their study they have shown that NPs in the range of 1–10 nm can act differently against Gram- bacteria by (i) attaching to cell membrane affecting permeability and respiration (ii) penetrating inside bacteria and damage them or (iii) via releasing silver ions. Gurunathan et al. have shown the antibacterial capacity of AgNPs of about 5 nm diameter against Gram- and Gram+ bacteria (Gurunathan et al., 2014).

Properties of AgNPs have also been investigated in TERM mostly for wound dressing applications. Wounds such as chronic wounds and burn wounds are highly prone to infection. Moreover, increased incidence of multi-drug resistant microbes related infections are a challenging health problem and requires the incorporation of antimicrobial components in the scaffold design for wound healing. These particles have been incorporated in scaffolds made of different materials e.g., poly(vinyl alcohol) (PVA), PCL, gelatin, chitosan-alginate and cellulose acetate scaffolds. All the resulting scaffolds exhibited strong antimicrobial activity.

NPs can be formed before the incorporation in the scaffolds and then loaded or a composite can be formed during which AgNPs are formed *in situ* in the structure using reducing agents (heat, UV, chemicals). These different scaffolds can be produced in different formats including bulk materials, electrospun fibers, fibers mats, nanofibrous or porous scaffolds (Son et al., 2004; Hong et al., 2006; Rujitanaroj et al., 2008; Augustine et al., 2016; Bhowmick and Koul, 2016; Mokhena and Luyt, 2017; Pankongadisak et al., 2017; Rosa et al., 2017; Santos et al., 2017; Venkatesan et al., 2017; Biswas et al., 2018; Mehrabani et al., 2018; Yahyaei et al., 2018). In an animal model, Tian et al. studied the impact of AgNPs treatment on burn and diabetic wounds as potential wound healing accelerator (Tian et al., 2007). They found that the delivery of AgNPs not only had an antimicrobial effect but also it has accelerated the rate of healing. Male BALB/c Mice with deep partial-thickness burn wounds normally cured after 35.4 ± 1.29 days. Wounds treatment with silver sulfadiazine (SSD) had prolonged the healing period to 37.4 ± 3.43 days. AgNPs, in contrast, have enhanced the healing process to 26.5 ± 0.93 days (Figure 3B). In the diabetic wounds, a similar effect has been observed and wound treated with AgNPs were healed 16 ± 0.41 days after injury, while wound treated with SSD required 18.5 ± 0.65 days for complete healing. They also have discovered that AgNPs had the ability to regulate the cytokines associated in burn wound healing. Significant decrease in neutrophils was found in wound treated with AgNPs compared to SSD groups which indicate effect of AgNPs to decrease the local and systemic inflammatory response. This effect can be complemented with hydrogels as hydrogels can prevent contraction of the wound due to high water uptake content. By Wang et al. AgNPs poly(gamma-glutamic acid) (⋎-PGA) hydrogel copolymer has been shown to promote wound healing *in vivo* on male BALB/c mouse compared to control groups (Wang et al., 2018). Collagen deposition and intact epidermis layer formation have been observed after 14 days of impaired wound healing with histological analysis.

In the same area, AgNPs have been utilized to elaborate chitin/nanosilver composite antimicrobial scaffolds. It has been also observed that the blood clotting efficiency was increased with this scaffold due to the fact that silver can affect the pathway of coagulation by denaturing the anticoagulant proteins (Madhumathi et al., 2010). AgNPs (100 nm) have also shown to enhance the biocompatibility and structural stability of decellularized porcine liver through crosslinking (Saleh et al., 2018). Its crosslinking efficiency was appraised and compared to that of glutaraldehyde and ethyl carbodiimide hydrochloride and N-hydroxysuccinimide. Enhancement in the ultra-structure of the collagen fiber in decellularized liver and slower *in vitro* degradation have been observed compared to control groups. It is worthy to mention that AgNPs also can show cytotoxic effect over cancer cells. It was reported that a porous chitosan-alginate with biosynthesized AgNPs has been shown to have cytotoxic effects against MDA-MB-231 breast cancer cells (Venkatesan et al., 2017). These examples show the potential of AgNPs for TERM.

## Ceramic Nanoparticles

Ceramic nanoparticles (CNPs) are basically comprised of inorganic compounds, besides metals, metal oxides, and metal sulfides and they can be used in production of nanoscale materials of various shape, size, and porosity (Singh et al., 2016). IN general, CNPs can be classified according to their tissue response as being inert, bioactive or resorbable ceramics and magnetic NPs (Kohn, 2003).

### Bioactive Glass Nanoceramics

Bioactive glass ceramic nanoparticles (n-BGC) with SiO_2_-CaO–P_2_O_5_-Na_2_O core structures were established by Larry Hench's team in 1969 (Jones, 2015). Bioglasses can be formed from various elements such as silicone, sodium, potassium, magnesium, phosphorous, oxygen, and calcium which can be absorbed by the cells (Taygun and Boccaccini, 2011). Various techniques have been developed to produce nanoscale bioactive glasses such as microemulsion, laser spinning, sol-gel, and gas-phase synthesis (Taygun and Boccaccini, 2011). nBGC can provide faster ion release compared to bulk bioactive glasses due to their improved specific surface area; consequently, enhancement of bioactivity and adsorption of proteins can be expected (Boccaccini et al., 2010). Effect of various morphologies of CaO–P_2_O_5_-SiO_2_ based nBGC on their bioactivity was investigated through utilization of lactic acid (LA) in the sol–gel procedure through immersion in simulated body fluid (Chen et al., 2009). Addition of LA resulted in a decrease in the size of bioactive glass nanoparticles (nBGs). nBGs of either unimodal or bimodal (narrow) pore size distribution had higher bioactivity compared to the nBGs with smooth surface morphology. Wang and colleagues studied nBGs with a diameter of 12 nm (BP-12) instead of mixing conventional bioglass (diameter of 200 nm) with gelatin to manufacture a simple hydrogel for wound dressing application (Wang C. et al., 2016). Composition of gelatin with BP-12 could provide hydrogel with pronounced thixotropy (becoming less viscous) characteristic at a practically usable shear rate. Such a polymer-colloid mixture can be in a gel state, and become injectable under shear, and return to gel state as settled again; therefore, its use becomes easy for wound coverage. Fast tissue formation including regeneration of cutaneous-tissue was observed within 7 days after implantation in rats.

Antibacterial and angiogenic properties and excellent bioactivity of nBGs have made them as a suitable candidate for dentin regeneration applications. Incorporation of boron modified nBGs in the cellulose acetate/oxidized pullulan/gelatin-based constructs has shown promising results for dentin regeneration through increase in cellular viability, Intracellular calcium deposition (ICD) and ALP activity while keeping the boron ion released below toxic level (Moonesi Rad et al., 2019). Rad et al. demonstrated that incorporation of 6.25 mg/ml boron doped nBGs into cell culture media has increased the ALP activity and ICD. The group also showed human dental pulp stem cells' odontogenic differentiation were enhanced by immunocytochemical staining of dentin sialophosphoprotein, osteopontin, and collagen I (Rad et al., 2018).

nBGs are also attractive candidates for bone tissue engineering applications. In a study by Covarrubias et al. it was reported that incorporation of dense nBGs into chitosan-gelatin polymer blend has promoted the higher activity of alkaline phosphatase compared to mesoporous bioactive glass nanosphere composites (Covarrubias et al., 2018). *In vivo* experiments have shown that in dense chitosan-gelatin hydrogels containing 5% bioactive glass nanoparticles had the highest amount of new bone formation (~80%) in the defect area after 8 weeks of implantation compared to control groups. In another study, multifunctional poly(citrate-siloxane) (PCS) elastomer based nBGs were developed for bone tissue engineering (Li Y. et al., 2018). Hybrid material showed intrinsic biomineralization with photoluminescent properties. Addition of nBGs has increased elastomeric modulus of PCS from 20 to 200 MPa. Biodegradation and *in vivo* metabolization of the nanocomposite has been tracked through real-time monitoring through exciting with blue fluorescence at the 365 nm thanks to inherent and high photoluminescence quantum yield and lifetime of PCS. This nanocomposite had proliferative effect on osteoblasts (MC3T3-E1). Additionally, it enhanced osteoblastic differentiation of these cells and has also decreased the *in vivo* inflammatory response toward the biomaterial. Various other applications have been investigated using nBGs for bone tissue engineering including copper containing nBGs in gelatin coated scaffolds (Zheng K. et al., 2018), miRNA delivery with nBGs with ultralarge pores which leads to highly efficient miRNA loading (Xue et al., 2017), and osteogenic differentiation induction of adipose-derived stem cells using monodispersed nBGs (Guo Y. et al., 2018).

### Bioresorbable Nanoceramics

Bioresorbable nanoceramics have calcium phosphate (CaP) based composition which include variety of materials such as hydroxyapatite (HAp), calcium aluminate, tricalcium phosphate, calcium phosphate dicalcium phosphate dehydrate, calcium carbonate (CaCO_3_), calcium sulfate hemihydrate, octacalcium phosphate and biphasic calcium phosphate. HAp is major component (inorganic) of natural bone and under neutral or alkaline conditions it is the most stable form of phosphate salts. These materials have been applied in orthopedics such as bone substitutes (Yao et al., 2017). Various manufacturing processes including chemical synthesis methods have been developed for the production of HAp NPs with precise control over the nanostructure (Ferraz et al., 2004; Sadat-Shojai et al., 2013). It is difficult to synthesize highly pure HAp as calcium phosphates have variety of derivatives and reaction conditions plays crucial role in the synthesis of CaPs and their properties (Han et al., 2006). Combination of different methods can be utilized to for enhancing properties of final product (Sadat-Shojai et al., 2013). Various ions can be incorporated to the HaP lattice to modulate the characteristic features of the scaffold for desired TERM application such as degradation properties and cellular responses. Cadmium, silicon, yttrium, silver, zinc, copper, magnesium, and trace elements have been used for modifying the HaP properties (Ergun et al., 2002; Cox et al., 2014; Hidouri et al., 2018). Various single ion substitutions in HAp were reported before (Lin and Chang, 2015; Fihri et al., 2017; Kim et al., 2018; Pal et al., 2019).

Composite scaffold bearing HAp NPs have shown different mechanical properties which mainly depend on the nature of composite and preparation method. Incorporation of HAp NPs has been reported to increase modulus of compression in the porous shape memory polyurethane (Yu et al., 2018) or enhance the ultimate compressive strength in PCL/poly(lactic-co-glycolic acid) (PLGA)/HAp NPs (Li X. et al., 2017). In another study compressive modulus of gelatin HAp NPs composite hydrogel has been shown to decrease by increasing the weight percentage of HAp NPs. Such decrease was attributed to negative effect of HAp NPs on the crosslinking efficiency (Raucci et al., 2018).

HAp is commonly used in TERM applications. Combination of HAp with various from of carriers such as electrospun fibers (Cai et al., 2017; Samadian et al., 2018), porous scaffolds (Guo M. et al., 2018), and hydrogels (Ghosh et al., 2017) have been reported for preparation of nanocomposite materials to modulate the desired cellular activities. Recently, graphene oxide-incorporated silicate-doped nano-HAp composites have been used for reinforcement of fibrous scaffolds of PCL produced by wet electrospinning for bone tissue engineering (Dalgic et al., 2018). Silicate-doped nano-hydroxyapatite (10%)—graphene oxide (4%) group was reported to enhance the adhesion, spreading, proliferation and ALP activity of Saos-2 cells compared to other scaffold groups. Qian et al. compared the behavior of a biomimetic PLGA scaffold with and without HAp NPs. NPs were reported to enhance cellular activities including cell attachment, proliferation, and differentiation of pre-osteoblastic cells (Qian et al., 2014).

**Figure 4:**
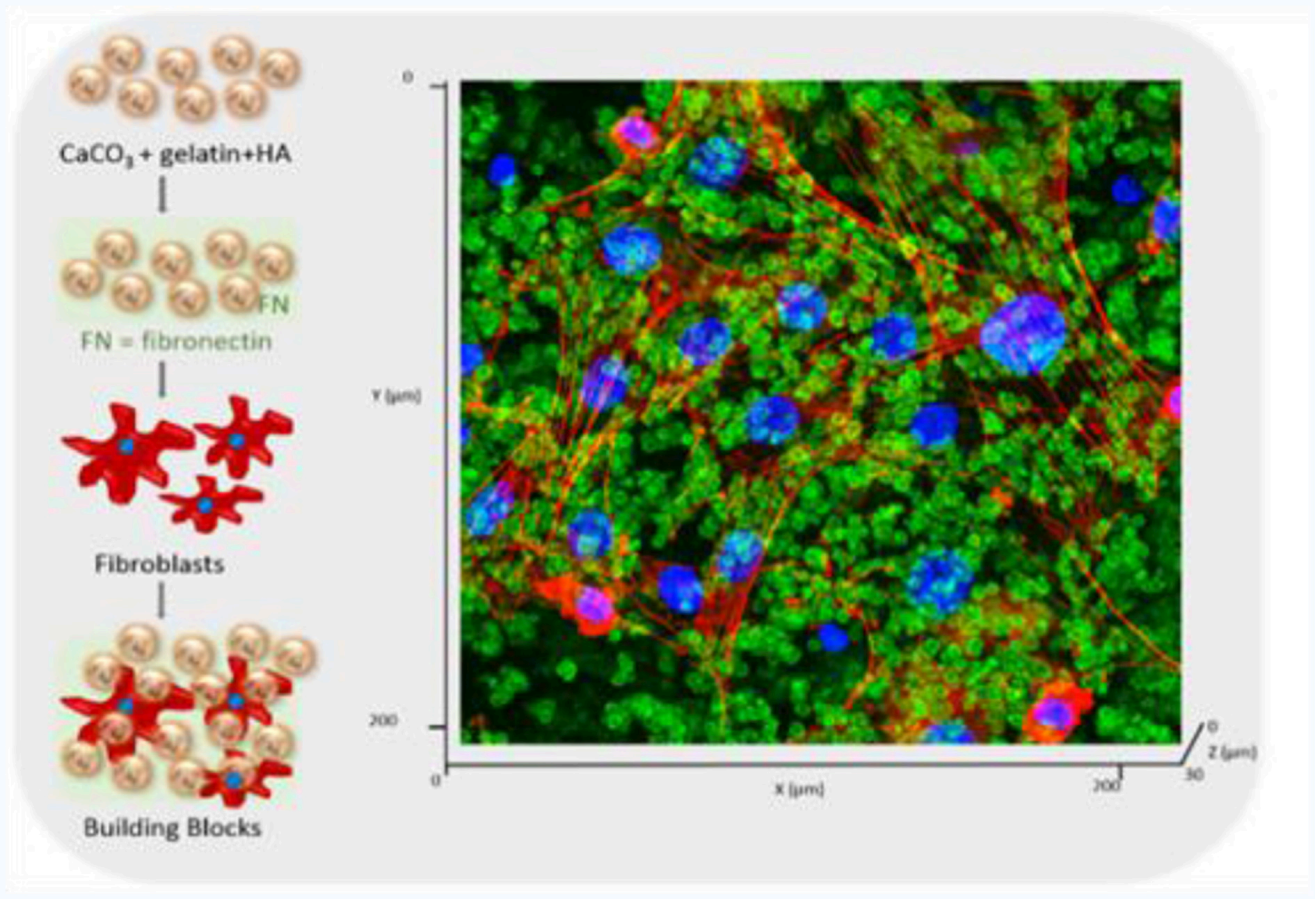
Development of a hybrid CaCO_3_ particular system doped with ECM components (gelatin, hyaluronic acid, fibronectin) for creating a building block strategy. Confocal images of the after 3 days of culture. The particles are labeled in green (stained by HA-FITC) and the fibroblasts were visualized with Rho-Phalloidin (red) and nuclei (blue). Reprinted with permission from Affolter-Zbaraszczuk et al. (2017).

Multi doping may also improve the properties of HAp and such substitutions are gaining huge attention nowadays. Ba^2+^ and Ho^3+^ ions as contrast agents for computed tomography (CT) have been doped into nHAp via microwave-assisted synthesis and under various operating voltages significant enhancement in the contrast efficiency was observed (Zheng X. et al., 2018). Application of co-doped HAp NPs in bone tissue engineering has focused to enhance cellular activity including attachment, proliferation and differentiation. Alshemary et al. (2017) have successfully synthesized ferric (Fe^3+^)/selenite (Se${}_{4}^{2-}$) co-doped HAp materials via microwave refluxing procedure. Degree of crystallinity was decreased and crystallite growth was inhibited by increasing the amount of Fe^3+^ doping. Acceleration in growth of apatite layer and increase in degradation rate of HAp were reported by introducing dopants. Cytocompatibility and favorable osteoblastic differentiation of stem cells were observed in Fe-SeHAp scaffolds. The group proposed Fe-SeHAp group as a suitable bioceramic for bone tissue regeneration. Recently, our group developed hybrid microspheres for constructing a CaCO_3_ particles that are simple to administer and will allow to build-up various tissues. These particles are based on ECM factors such as fibronectin, hyaluronic acid and gelatin which can form a system for TERM applications such as wound management, as shown in Figure 4. Furthermore, these building blocks will provide stepwise build-up to manufacture stratified 3D cellularized scaffolds (Affolter-Zbaraszczuk et al., 2017).

CaPs have been utilized with different types of polymers to produce nanocomposite materials (Vieira et al., 2017). Advantages of these nanocomposites such as excellent mechanical characteristics could be utilized for bone tissue regeneration through enhancing scaffolds' performance (Vieira et al., 2017). Ataol et al. synthesized nano calcium phosphate particles (CaP NPs) using flame spray pyrolysis technique (Ataol et al., 2015). Urine-derived stem cells treated with the CaP NPs had shown elevated ALP activity compared to control cells, demonstrating osteogenic differentiation of these cells. From the above mentioned articles and vast number of the conducted researches in the literature it can be deduced that addition of CaP family of materials in the form of NPs can provide a better osteogenic differentiation for TERM applications.

### Bioinert Nanoceramics

**Figure 5:**
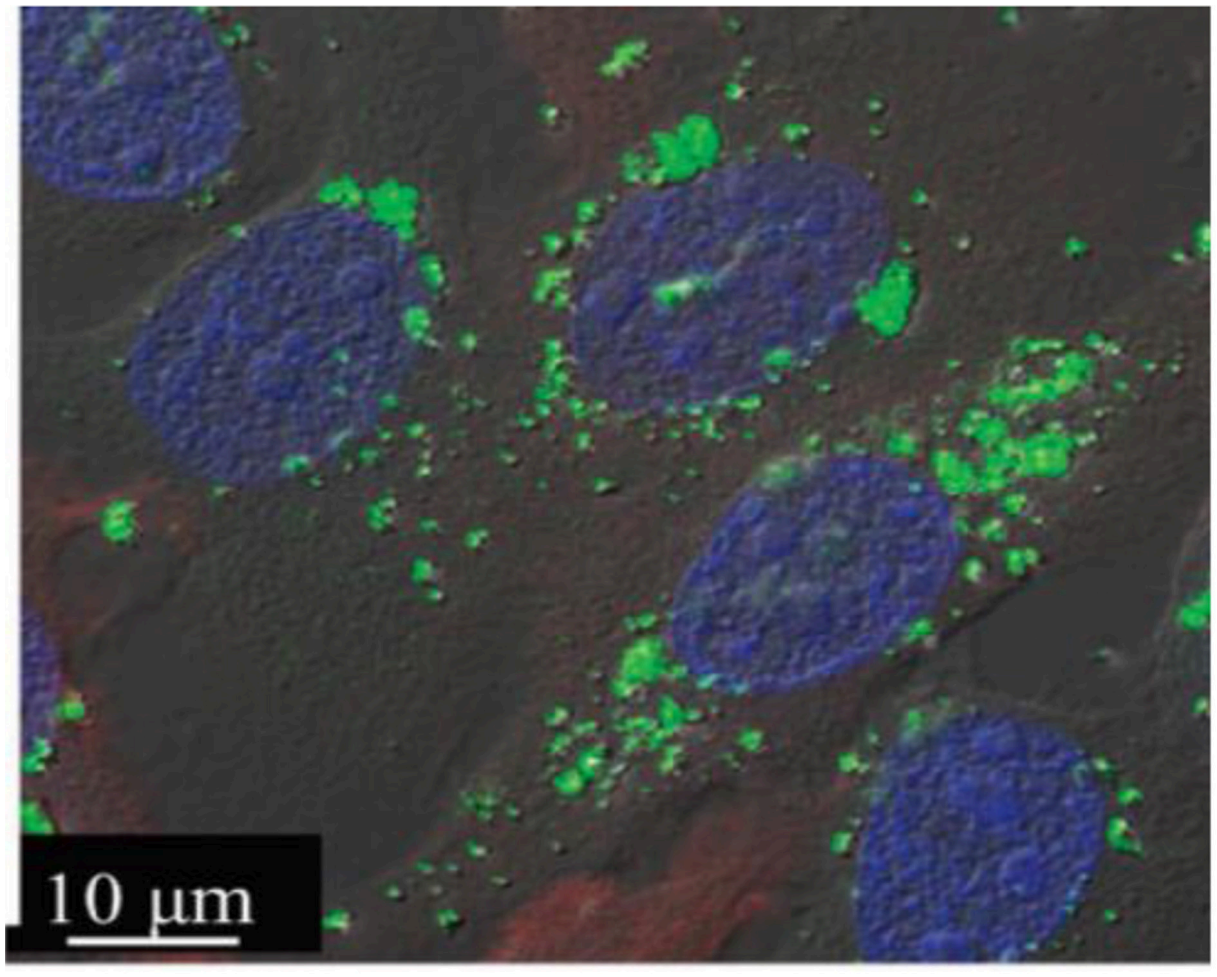
A merged fluorescence confocal image of FMN–MTNs (green dots) in BT-20 cancer cells where the nuclei were stained with DAPI (blue spots). Reprinted from Wu et al. (2011) with permission from The Royal Society of Chemistry.

Bioinert nanoceramics including titanium dioxide (TiO_2_), zinc oxide (ZnO) are utilized for different medical applications as they show positive interactions with body tissues. TiO_2_ NPs can be synthesized with different manufacturing processes including hydrothermal, solvothermal, sol-gel process and emulsion precipitation methods (Vollath et al., 1997; Zhao et al., 2007; Gupta and Tripathi, 2011). Zhao et al. have prepared TiO_2_ NPs by flame synthesis (Zhao et al., 2007). By using this method it is possible to manufacture uniformly distributed (in size) bioceramics in targeted size range. With the advancement of nanotechnology, TiO_2_ nanoparticles, nanotubes or nanoprobes labeled with the fluorescent dye or magnetic resonance contrast agents have been successfully prepared for cell imaging through fluorescent analysis or magnetic resonance imaging (MRI) (Fei Yin et al., 2013). Mesoporous titania nanoparticles (MTNs) with superior biocompatibility (LC50 ≈ 400 μg mL^−1^) and a functionalized MTNs with a phosphate-containing fluorescent molecule (flavin mononucleotide; FMN) have been recently synthesized. FMN-MTNs were used as a satisfactory intracellular bioimaging agent (Wu et al., 2011). BT-20 cells were incubated with FMN-MTNs for 4 h, and cytoplasm of cells was visualized as the FMN-MTNs emitted green fluorescence. Observing such emission indicates the presence of FMN molecules in large surface area (ca. 237.3 m^2^ g^−1^), and they were further observed inside the MTNs without much leaching (Figure 5). The mesoporous TiO_2_ have been applied with magnetic-targeting for dual-modal imaging and photodynamic therapy through combining NIR mediated photodynamic therapy, chemotherapy and gene therapy in a synergy manner for cancer treatment (Yu et al., 2017).

Various methods were developed for synthesizing nanoscale ZnO powders including precipitation, hydrothermal synthesis, spray pyrolysis, thermal decomposition and electrochemical growth (Vollath et al., 1997; Padmavathy and Vijayaraghavan, 2008). Various parameters such as type of solvent and precursor, pH and reaction temperature can affect the particle size of ZnO. Padmavathy and Vijayaraghavan used precipitation and the base hydrolysis methods for manufacturing nanosized ZnO (Padmavathy and Vijayaraghavan, 2008). Both methods yielded ZnO particles with 10–50 nm size range. Both NPs were reported to demonstrate better antibacterial properties compared to bulk ZnO. In another study, biological technique was used for synthesis of ZnO NPs through rapid, single step and green synthesis process by utilizing *Sargassum muticum* which is a brown marine macroalga (Azizi et al., 2014). Based on the Fourier transform infrared (FTIR) spectroscopy analysis, polysaccharides in *Sargassum muticum* extracts were reported to be involved in the production of ZnO NPs.

Similar to AuNPs and AgNPs, metal oxide NPs can be utilized as antimicrobial protection agents in the context of TERM (Laurenti and Cauda, 2017). Recently, nanocomposite of chitosan/hydroxyapatite-zinc oxide (CTS/HAp-ZnO) supporting organically modified montmorillonite clay (OMMT) has been manufactured for bone tissue engineering applications (Bhowmick et al., 2018). Nanocomposite has shown strong antibacterial activities when facing Gram+ and Gram- bacteria. Mechanical properties of nanocomposite and proliferation of osteoblastic MG-63 cells on this biomaterial have shown improvement compared to control group. In another bone tissue application, a hybrid scaffold composed of polyurethane nanofibers that was reinforced with zinc oxide-functionalized multi-wall carbon nanotubes has been developed (Shrestha et al., 2017). Electrospun scaffolds with 0.2 wt% ZnO, and 0.4 wt% functionalized multi-wall carbon nanotubes were found to exhibit antibacterial activity and cytocompatibility.

### Magnetic Nanoparticles

Magnetic nanoparticles (MNPs) are iron oxide NPs, (usually Fe_3_O_4_ or Fe_2_O_3_) which are widely studied in biomedical field because of their lower toxicity. Co-precipitation is used as most conventional method for synthesizing Fe_3_O_4_ or γ-Fe_2_O_3_ (Gupta and Gupta, 2005). In this method ferric and ferrous ions in highly basic solutions are mixed (1:2 molar ratio, respectively) at room temperature or at high temperatures. Similar to ZnO NPs, Fe_3_O_4_ NPs were reported to be synthesized from *Sargassum muticum* (Mahdavi et al., 2013). The aqueous extraction of seaweed and ferric chloride solution were mixed to produce Fe_3_O_4_ NPs. Xiaoming et al. reported that MNPs have been extensively utilized in MRI applications (Li et al., 2016). It consists of various applications like imaging cancer cells, pursuing stem cells *in vivo* and monitoring of transplanted tissues. In this context, they can also be used for monitoring of engineered tissues. Moreover, superparamagnetic magnetite nanocrystal clusters (SMNC) can be utilized for cell imaging. Mini emulsion/sol-gel and polyol techniques were used for synthesizing SMNCs. They were prepared by coating with polyetherimide, citric acid or silica. These SMNCs possessed very high sensitivity toward magnetic resonance, and had no adverse effect on cell viability (Li et al., 2016). It has been reported that NPs with small size (< 100 nm) and narrow size distribution are suitable for both *in vitro* and *in vivo* biomedical applications (Medeiros et al., 2011). However, this optimum size can be changed depending on the applications especially for multi-functional applications including combined drug targeting and imaging. Concerning the *in vitro* and *in vivo* applications, the super paramagnetic behavior of MNPs is also of interest as they lose magnetism after removing magnetic field. Retaining of magnetism is related to the size of particles. NPs with 10–50 nm size and proper surface coating can have long circulation times, and they can also manipulated by an external magnetic field (Medeiros et al., 2011).

One of the exciting applications of TERM is the neuroregeneration and nerve tissue engineering which could improve life quality of the patients with nerve injuries. Various studies have been conducted to deliver growth factor conjugated MNPs to cells, monitoring the fate of MNPs or stimulate the cells with MNPs (Alon et al., 2015; Marcus et al., 2015; Zuidema et al., 2015; Giannaccini et al., 2017; Willmann and Dringen, 2018). Recently, superparamagnetic iron oxide (SPIO)-Au core-shell NPs decorated with nerve growth factor (NGF) with low toxicity have been developed for neuron growth and differentiation (Yuan et al., 2018). NGF functionalized NPs have provided higher neuronal growth and orientation on PC-12 cells under dynamic magnetic fields utilizing rotation have been obtained compared to static magnetic fields. Magnetic NPs also have been used for controlling collagen fiber orientation dynamically and remotely *in situ* during the gelation period through an applied external magnetic field (Chang et al., 2017). Magnetically activated 3D gels bearing neurons showed natural cellular viability and electrical activity with elongated, co-oriented morphology. The iron oxides also have the ability to pass blood brain barrier, it could be used for conjugation of various peptide and growth factors to cure and regenerate brain tissue (Pilakka-Kanthikeel et al., 2013). In a study, Fe_2_O_3_ was used for conjugation of peptide antisauvagine-30 (ASV-30) to reduce anxiety-like behavior of rats through binding to corticotropin releasing factor type 2 receptors (Vinzant et al., 2017). *In vivo* results demonstrated that systemic application of iron oxide+ASV-30 decreased anxiety (due to amphetamine withdrawal) with no impact on locomotion.

## Polymeric Nanoparticles

For polymeric nanoparticles (PNPs), formulation, size, shape, surface chemistry and charge, porosity, mechanical strength, solubility, degradation rate and so on are among the features which can be adjusted for versatile purposes in TERM. Low cytotoxicity of PNPs, good biocompatibility, higher permeation and retention (EPR) effect, ability to deliver poorly soluble drugs and sustained release of them, retaining bioactivity of bioactive agents from enzymatic degradation for tissue engineering applications make PNPs as one of the fastest growing platform to overcome obstacles in TERM. Most of the new PNPs systems are designed to be sensitive to different physicochemical stimuli such as magnetic field, temperature, enzymes, pH, light, reducing/oxidizing agents which helps delivery or targeting systems with high specificity and efficiency for TERM applications (Cheng et al., 2013; Lale et al., 2014; Tang et al., 2016).

### Composition, Manufacturing Process and Structure of Polymeric Nanoparticles

**Figure 6:**
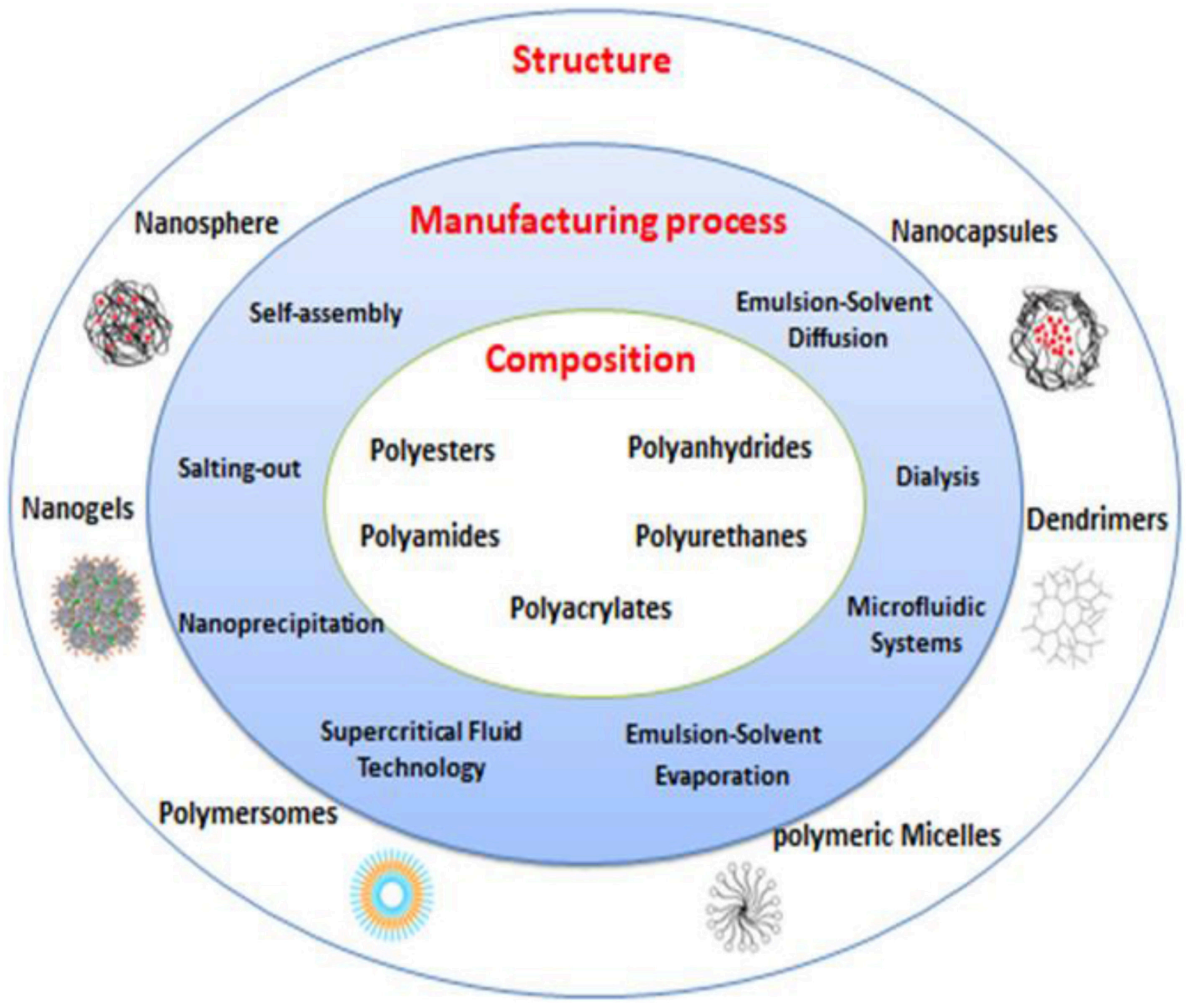
Different polymeric nanoparticles (PNPs) based on composition, manufacturing process, and structure.

Classification of PNPs can be based on different criteria such as composition, structure and manufacturing process (Figure 6). To achieve desired optimum properties, various PNPs have been designed using biodegradable and biocompatible natural and synthetic polymers. Polysaccharides (dextran, heparin, alginate, chitosan, hyaluronic acid, pullulan), proteins (albumin, gelatin, elastin, silk) and synthetic polymers such as polyesters, polyamides, polyanhydrides, polyurethanes, polyacrylates have been used alone or in conjugation with specific functional moieties or in combination with other materials to provide functionalities in TERM field (Nicolas et al., 2013; Hudson and Margaritis, 2014; Elsabahy et al., 2015; Bhatia, 2016); (Knopf-Marques et al., 2016).

PNPs can be manufactured in different shapes such as nanospheres, nanocapsules, polymersomes, dendrimers, polymeric micelles, and nanogels. Morphology of NPs will govern their macroscopic behavior; therefore, depending on the application, choosing suitable morphology is a critical point in manufacturing NPs (Nicolas et al., 2013; Bhatia, 2016); (Tang et al., 2016).

Different methods have been established for preparation of NPs such as various forms of emulsification (single and double emulsion/solvent evaporation, spontaneous emulsification, emulsion/solvent diffusion, emulsion polymerization), self-assembly, supercritical fluid (SCF) technology, nanoprecipitation, salting-out, dialysis, and microfluidic systems (Hasani-Sadrabadi et al., 2015; Bhatia, 2016; Tang et al., 2016; Herranz-Blanco et al., 2017).

### Delivery of Bioactive Agents

Controlled delivery of bioactive compounds in TERM relies on the success of retention of the delivery vectors in the vicinity of regenerating tissue (Veiseh et al., 2015; Wang Q. et al., 2016). Designing a reproducible ECM which can resemble the complex nature of native tissue microenvironment with comparable mechanical strength can be obtained through choosing appropriate 3D scaffold design with incorporation of bioactive agent carriers (Barthes et al., 2015; Affolter-Zbaraszczuk et al., 2017). In recent years, to improve delivery of bioactive agents in the sense of loading, targeting, and efficacy, NP based systems have been changed from simple delivery to multifunctional responsive systems. Moreover, hybrid systems have been developed for integration of NPs within scaffolds for controlled release (Santo et al., 2010). The tailorable properties of PNPs provide versatile paths for designing NPs through applying chemical and physical techniques to optimize controlled delivery. It is crucial to control microenvironment of cells through physiochemical stimuli to anticipate and control behavior of cells in tissues. Introducing stimuli such as proteins and drugs into milieu of cells in the form of encapsulated NPs alone or embedded into delivery scaffolds such as hydrogels, fibers, foams, and so on, can provide the stable signals for cellular activities. Due to complex architecture of tissues such as different cell types and multicomponent extracellular matrix, it is inevitable to use nanoscale systems for delivering bioactive factors; especially for multiple growth factors delivery in sequential and time dependent manner. Ability to tune release behavior from both PNPs and scaffolds through modifying composition, bulk and surface chemistry can provide delivery systems with spatiotemporal adjustable character suitable for targeted tissue (Chen et al., 2010; Jeon et al., 2013; Pérez et al., 2013); (Gaharwar et al., 2014).

Recently, plasma protein based NPs have gained attention due to their high bioavailability, non-toxicity, biodegradability, ease of manipulation, long *in vivo* half-lives and long shelf lives. There are more than 100,000 proteins in human plasma, but just a couple of these proteins have been used in TERM as a nanocarrier platform for imaging, drug delivery and tissue regeneration (Tezcaner et al., 2016). High density lipoproteins (HDL) NPs are among candidates for enhancing photodynamic therapy applications through presenting excellent tumor targeting and internalization capacity (Wang Y. et al., 2016; Raut et al., 2018). NPs from albumin, as the most abundant plasma protein, were used for bone regeneration through sustained release of bone morphogenetic protein-2 (BMP-2) (Wang Z. et al., 2016). Degeneration of the intervertebral disc (IVD) can happen due to several reasons such as enhanced local MMPs expression decreased quantities of ECM components which could lead to chronic lower back pain (Tong et al., 2017). IVD regeneration can be promoted using stem cell migration to the defect site. Zhang et al. have prepared albumin/heparin NPs as an injectable carrier for encapsulation of stromal cell-derived factor-1α (SDF-1α) as chemoattractant for the homing of bone marrow resident MSCs (Zhang et al., 2018). *In vitro* results have shown dose-dependent effect of SDF-1α over migration of the MSCs (at 50 and 100 ng/ml SDF-1α concentration, 41 and 64% MSCs migration after 24 h, respectively). Better regeneration compared to SDF-1α alone (control group) has been obtained as demonstrated by histological analysis, mRNA and protein levels for collagen type II, SOX9 and aggrecan.

Fibrin as another plasma protein has been used for encapsulation of VEGF for promoting the angiogenesis for wound healing applications (Mohandas et al., 2015). However, there are limited studies on plasma proteins. Disadvantages such as adverse immunological reaction, disease treatment, batch to batch variation or expensive isolation can be encountered in natural based materials; but plasma proteins have huge research potential as nanocarriers for TERM applications.

Bone tissue related diseases such as tumor or trauma generally are treated with bone grafts and substitutes. Nowadays TERM has provided an alternative approach for bone tissue regeneration through offering a variety form of 3D scaffolds. Bone scaffolds containing stem cells have the advantage of controlling the cellular activity such as differentiation, if appropriate bioactive agents such as drugs (e.g., dexamethasone) or growth factors [e.g., bone morphogenetic proteins (BMPs)] are incorporated in them (Basmanav et al., 2008; Santo et al., 2015; Wang et al., 2015). The ability to adjust degradation rate depends on physiological pH and intracellular drug release has led to manufacture of vast types of polymeric micelles as one of the widely used type of NPs. Santo et al. have designed a biodegradable pH responsive micelle from gelatin grafted with lactic acid oligomers and encapsulated dexamethasone (dex) for investigation the effect of intracellular release of dex for bone tissue regeneration (Santo et al., 2015). Efficient mineralized bone tissue formation was observed inside gelatin hydrogels containing dex loaded micelles seeded with pre-cultured rat bone marrow stem cells up to 4 weeks after implantation in rat ulna. In a work by Yilgor et al. how single or sequential or simultaneous growth factor delivery of BMP-2 in PLGA and BMP-7 in poly(3-hydroxybutyrate-co-3-hydroxyvalerate) (PHBV) nanocapsules in 3D chitosan and chitosan—poly(ethylene oxide) (PEO) fiber mesh constructs was studied (Yilgor et al., 2009). It was reported that rat bone marrow MSCs seeded on chitosan scaffolds which contains NPs providing sequential delivery release profile of BMP-2 (fast release) and BMP-7 (slow release), had the highest ALP activity per cell compared to those that interact with NPs loaded with one type of BMP or with those that provide the simultaneous release of two BMPs. The group reported that for sequential delivery, NPs adhered on the fibers demonstrated better than the NPs which were embedded within fiber structure.

Temperature sensitive poly(N-isopropylacrylamide) (PNIPAM) NPs have been developed for slow VEGF delivery within the collagen hydrogels (Adibfar et al., 2018). Endothelial differentiation of bone marrow derived MSCs and tube (capillary-like) formation in the hydrogels after 14 days of incubation in the osteogenic medium were observed. Expression of osteocalcin, collagen type I and runt-related transcription factor 2 as osteogenic markers besides the expressions of kinase insert domain receptor, von Willebrand factor and platelet-endothelial cell adhesion molecule-1 as angiogenic markers promoted the vasculature within bone tissue engineered constructs. Chen et al. have developed a conductive poly(aniline) NPs *in situ* in poly(L-lactide) PLLA/tetrahydrofuran through polymerization/thermal induced phase separation (TIPS) technique to produce PLLA based nanofibrous construct with conductivity feature for bone TE (Chen J. et al., 2018). Presence of well-distributed poly(aniline) NPs provided scaffolds with conductivity close to natural spongy bone. Higher proliferation, higher mineralization and osteogenic differentiation were observed on conductive scaffolds compared to control groups.

Angiogenesis as a key factor in TERM prevents cell necrosis in 3D scaffolds through providing nutrients, wastes and gas exchange. Delivery of angiogenesis triggering factors such as VEGF can enhance formation of blood vessels and promote tissue healing process. Various studies have been conducted for delivery of bioactive agents such as growth factors for promoting the neovascularization *in vitro* and *in vivo* (Lee et al., 2017; Wang B. et al., 2017). Controlled delivery of VEGF with appropriate release profile plays an important role in vascularization. VEGF loaded PCL NPs incorporated Poly(L-lysine)/hyaluronic acid polyelectrolyte multilayer film system has been reported as an approach for controlled delivery of VEGF for angiogenesis (Vrana et al., 2014). Xie et al. have embedded platelet-derived growth factor-BB (PDGF-BB) loaded PLGA NPs within VEGF loaded chitosan and PEO electrospun nanofibers for mimicking and promoting natural skin healing process (Xie et al., 2013). *In vitro* studies conducted with adult human dermal fibroblasts have shown that a fast delivery of VEGF as angiogenesis promoter factor and PDGF-BB in delayed manner results in higher proliferation of fibroblasts. *In vivo* studies showed that use of nanoparticle/nanofiber scaffolds on rat skin resulted in faster wound healing compared to control groups due to increase in angiogenesis rate, re-epithelialization with quicker collagen deposition and earlier injury site remodeling.

Poor endothelialization and incomplete vascularization, can limit tissue-engineered scaffolds for regeneration of cardiovascular tissue damage. Tan et al. have established self-assembled NPs to accelerate vascularization of decellularized buffalo bovine jugular vein scaffolds through sustained release of VEGF (Tan et al., 2011). Low molecular weight heparin (LMWH) and N,N,N-trimethylchitosan chloride (TMC) which undergo self-assembly through non-covalent electrostatic interactions have been used to form NPs to protect bioactivity of VEGF. Results have demonstrated higher endothelial cell proliferation and new capillary formation, respectively. In another study, Izadifar et al. designed a bilayered NPs composed of PLGA as core and PLLA as shell polymers (Izadifar et al., 2016). They have used NPs of different compositions (PLGA and PLLA/PLGA bilayered) for obtaining sequential release of PDGF followed by co-release of VEGF and bFGF (basic fibroblast growth factor) as angiogenesis factors in fibrin matrix for cardiac tissue regeneration. *Ex vivo* angiogenesis using rat aortic ring assay has shown significant increase in the number of endothelial sprouts with maximum length of angiogenesis in sequential release group which was higher than that observed in simultaneous and only VEGF release groups.

**Figure 7:**
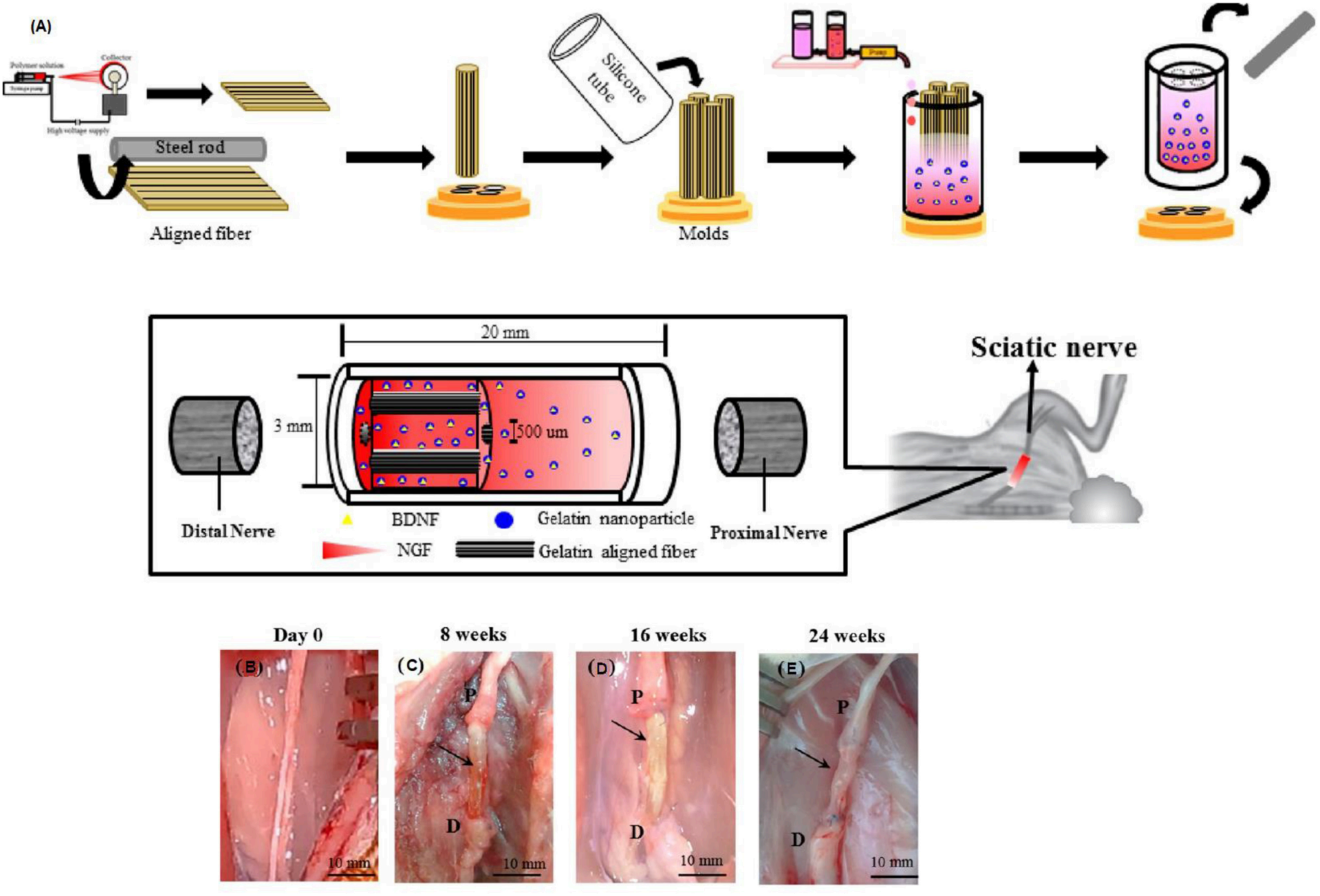
**(A)** Schematic illustration of fabrication process of nerve conduit with aligned fibers for axon outgrowth direction guidance and neurotrophic concentration gradient to attract the regenerated axon. **(B–E)** Nerve regeneration in the rabbit sciatic nerve transection model. **(B)** Sciatic nerve before surgery. **(C–E)** After surgical implantation of multichanneled scaffold combined with aligned nanofibers and neurotrophic gradient (MC/AN/NG) nerve conduit for 8, 16, and 24 weeks, the repaired nerve was clearly observed. The arrowhead indicates the regenerated sciatic nerve. (P, proximal end; D, distal end). Reproduced from Chang et al. (2017) with permission from American Chemical Society.

Neuroregeneration through delivery of bioactive agents and stem cells has provided new opportunities for treating central and peripheral nervous systems related diseases (Liu et al., 2018). Local delivery and spatiotemporal control over the chemokine SDF-1 through various NP based delivery system has gained lots of attention as the SDF-1 can help recruitment and migration of neural stem cells (Dutta et al., 2017; Zamproni et al., 2017). Nerve growth factor loaded chitosan NPs have been used for neural differentiation of canine MSCs (Mili et al., 2018). Coupling controlled delivery with spatiotemporal control over release of nerve growth factor with SDF-1 could be an alternative way to enhance the regeneration of central and peripheral nervous systems related injuries. IVD can cause low back pain and lead to disability. Teixeira et al. have used chitosan as a natural biodegradable and biocompatible polysaccharide and ⋎-PGA as a naturally occurring peptide containing of D- and L-glutamic acids to form self-assembled NPs for encapsulation of anti-inflammatory drug, diclofenac (Df) for intervertebral disc regeneration through inhibition of inflammatory processes (Teixeira et al., 2016). Df-NPs have shown reduced proinflammatory mediators [Prostaglandin E2 (PGE2), Interleukin 6 (IL-6) and IL-8] and reduced the expression of MMP 1 and 3, while enhanced aggrecan and collagen type II in a pro-inflammatory/degenerative bovine IVD organ culture. A model for peripheral nerve injuries has been developed by Chang et al. to enhance axonal regrowth and promote nerve healing (Chang et al., 2017). Natural biodegradable multichanneled scaffolds composed of ordered electrospun nanofibers with neurotrophic gradient has been designed to control axon outgrowth. Brain derived neurotrophic factor was encapsulated in gelatin NPs and nerve growth factor has been added freely into the scaffold. Neurotrophic gradient for both growth factors have been provided using gradient maker. Gelatin-based multichanneled structure is designed to mimic the nerve fascicular structure with appropriate mechanical properties (Figures 7A–E). Early delivery of nerve growth factor enhanced the initial stage of axon regeneration, while delayed release of brain-derived neurotrophic factor in encapsulated gelatin NPs augmented the late stage of myelination process. Differentiated neural stem cells effectively extended their neurites along the aligned nanofibers and higher cell density was observed in regions with high NGF concentration.

Complex process of wound healing makes the full reconstruction of functional skin a challenging task after injuries. Various NP based dressings have been developed for delivering bioactive agents with spatiotemporal control for enhancing the wound healing process (Berthet et al., 2017; Ghalei et al., 2018; Follmann et al., 2019). Recently, Kheradvar et al. have designed NP based delivery system for vitamin E due to its antioxidant activity, anti-inflammatory and scar-prevention properties for wound healing applications (Kheradvar et al., 2018). To do so, core-sheath nanofibrous system composed of silk fibroin/PVA/aloe vera and vitamin E containing starch NPs prepared by electrospinning method was incorporated into this system during preparation. With encapsulation efficiency of 91.63% for vitamin E, *in vitro* results have shown that, presence of NPs and aloe vera has increased cellular viability and cell-matrix interactions. However, vitamin E was more efficient in improving antioxidant activity compared to aloe vera.

Surface features play a critical role in biocompatibility features of biomaterials and strength of host immune system reactions. In a study, polymer model based particles were manufactured from degradable mesoporous silica template and they were used to study the effect of various surface-cell interactions without substrate dependency (Song et al., 2017). Poly(N-(2 hydroxypropyl)methacrylamide) (PHPMA), PEG and poly(methacrylic acid) (PMAA) were used to produce NPs within mesoporous silica template. Residual particles were removed after template crosslinking. After crosslinking the resulted NPs, contained only interfacial polymer, each type of NPs has been evaluated *in vitro* and *in vivo* for stealth properties. PEG NPs have shown better stealth properties compared to PHPMA and PMAA. PMAA NPs has shown fast elimination from plasma and quickly absorbed by the liver and high monocyte and macrophage association *in vitro* and *ex vivo*. Aggregated levels of particles (PMA and PHPMA) in the major organs of the mononuclear phagocyte system were comparable while they were ~2-fold higher than that of the PEG NPs. Similar systems can be applied for production of various NPs with similar structure but different type of materials and their biological response can be studied to select the most suitable candidate for various TERM applications.

### Imaging and Contrast Agents

Among different diagnostic tools, imaging can visualize diseased tissues, tissue healing and stem cell fate through providing contrast with respect to other tissues or by labeling NPs with an appropriate ligand. Fluorescent labeling, single photon emission computerized tomography (SPECT), ultrasound, MRI, NIR fluorescence imaging are among the widely used imaging techniques which alone or in conjugation with each other can provide sensitive, high resolution images from tissues through utilization of contrast agents (Harrison et al., 2014; Hong et al., 2014; Gu et al., 2017; Savla and Minko, 2017; Ekkelenkamp et al., 2018; Yan et al., 2018). Direct injection of contrast agents has poor outcomes due to short half-life, degradation, low concentration in desired tissue and toxicity at high dosages. Encapsulation of these imaging agents or attaching them on the surface of PNPs can provide longer half-life for circulation in body with efficient targeting in desired tissues.

Nowadays, most of visualizing and healing techniques have merged together for targeting the tumors by utilizing NPs with different moieties to act as both diagnostic and therapeutic tools. In TERM field it is also possible to label cells alone or within the scaffold to track the immune, differentiated and stem cells for disease treatment, tissue healing, cell migration and differentiation for monitoring applications. Molecular imprinted polymer (MIP) based NPs which are made through cell surface targeting molecular templates enable the construction of highly specific binding, 3D structures with affinity similar to antibodies for cell and tissue imaging. By mimicking glucuronic acid which is present in the form of hyaluronan on the surface of cells such as keratinocytes, Kunath et al. have imprinted glucurconic acid using (N-acrylamido) benzamidine (AAB) and methacrylamide (MAM) and polymerizable derivative of rhodamine as dye label for the detection of glycosylations on cell surface (Kunath et al., 2015). NPs applied on skin surface have shown localization of MIPs on the surface of keratinocytes without reducing cell viability. Detection of glycosylations on cell surfaces using MIP can provide a versatile synthetic tool to monitor compounds such as some glycanes which no natural receptors are available and decrease multiple labeling with various primary and secondary antibodies. Such MIPs can be also used for targeting of HA based scaffolds for monitoring scaffold behavior such as degradation.

Triggering of B cells' receptors by antigens causes priming of B and T cells and their differentiation to antibody secreting cells in the lymphoid organs. De Koker et al. have developed hydrogel NPs for antigen delivery in draining lymph nodes for immune cell subsets (De Koker et al., 2016). NPs were synthesized with infiltrating silica particles (mesoporous) with PMMA followed by disulfide-based crosslinking and Alexa Fluor 488-cadaverine (AF488) fluorescent labeling and template removal. PEGylated hydrogel NPs have shown higher lymph node targeting, consequently more dendritic and B cells turn into particle positive which followed by the priming of antigen-specific T cells. B cell activation has increased compared to non-PEGylated group. Similarly, modulating immune cells such as macrophages in TERM applications can provide more successful results through modulating immune responses such as inflammation especially for implant applications.

**Figure 8:**
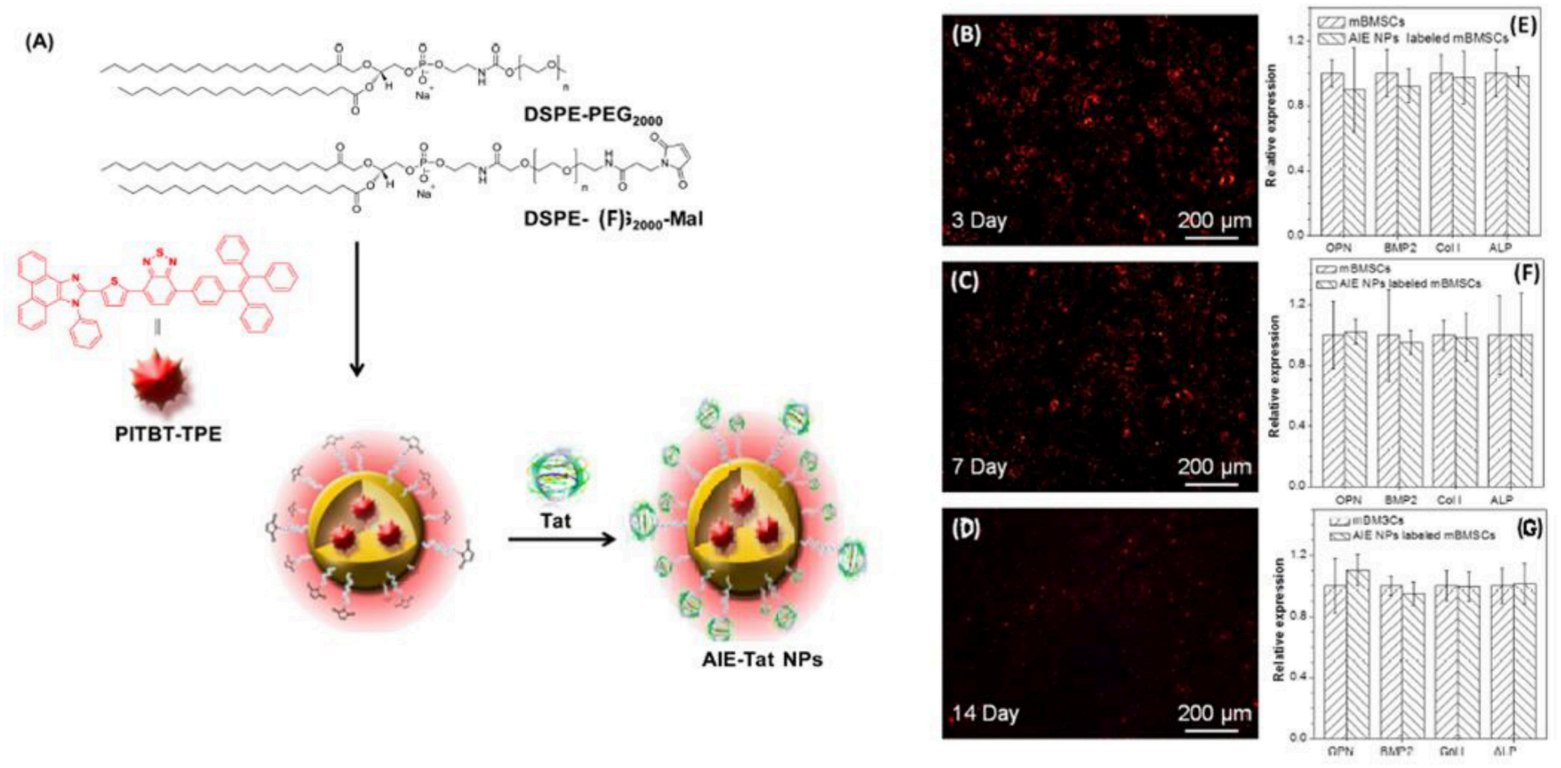
**(A)** Fabrication of AIE-Tat NPs based on PITBT-TPE Fluorogen (**B–D)** Fluorescent images of mouse bone marrow MSCs grown on an HA scaffold. **(E–G)** RT-PCR results of OPN, BMP2, Col I, and ALP gene expression in AIE-Tat NP-labeled and unlabeled mouse bone marrow MSCs after osteogenic induction for 3, 7, and 14 days. Reproduced from Gao et al. (2016) with permission from American Chemical Society.

Stem cells can be used for treatment of various tissues such as bone, but tracking the fate of stem cells requires non-invasive labeling methods. Gao et al. have developed aggregation-induced emission (AIE) fluorogen of benzothiadiazole-based emissive aggregate, PITBT-TPE, NPs labeled with cell penetrating Tat peptides (AIE-Tat NPs) for monitoring/tracking of bone marrow MSCs in HA scaffolds for monitoring bone regeneration in mouse (Gao et al., 2016). AIE-Tat NPs treated scaffolds have shown long term internalization with bright fluorescence all along osteogenic differentiation for 2 weeks (Figures 8A–G). In another study conducted by Wang et al. rhodamine- conjugated core-crosslinking biodegradable elastomer poly(glycerol-co-sebacate) acrylate (PGSAR) NPs were prepared and compared with rhodamine encapsulated NPs for long term stem cell tracking (Wang L. et al., 2017). *In vivo* studies conducted with mice have shown that methyl cellulose embedded rhodamine conjugated NPs had 28.29% of its initial fluorescence signal at day 28 whereas encapsulated rhodamine NPs had undetectable fluorescent signal after 7 days.

## Nanoparticles in Bioinks for 3D Printing

Currently 3D printing technologies have gained significant attraction in TERM field due to their superior control over design and manufacture of 3D scaffolds. Bioink as the main component of 3D bioprinting can be defined as a solution of one or more biomaterials which can be turned in to hydrogel form which encapsulate the required cell types. It can be stabilized or crosslinked during or after 3D printing through various mechanisms (Gungor-Ozkerim et al., 2018). Bioink based 3D printed scaffolds can provide a proper microenvironment for encapsulation of different cells within the hydrogels through modulating the biological, rheological and mechanical properties of scaffold (Gungor-Ozkerim et al., 2018). Integration of NPs within the 3D printed scaffolds can provide delivery bioactive agents for cells and tune mechanical strength of the scaffolds (Nowicki et al., 2017).

Synthetic polyurethanes (PU) have favorable mechanical properties, high biocompatibility, and tunable chemical structures which enable them to be used as 3D printing feeder for construction of custom made scaffolds. Hung et al. have used a synthetic biodegradable PU elastic NPs with hyaluronan (HA) as viscosity and cell aggregation enhancer, and Y27632 inhibitor as an alternative for transforming growth factor beta-3 (TGF-β3) for chondrogenic differentiation of MSCs as manufacture feeder materials (ink) for water based 3D printing for customized cartilage tissue engineering (Hung et al., 2016). *In vivo* implantation of scaffolds in rabbit knee with adipose-derived stem cells had resulted in better regeneration with significantly more type II collagen production compared to PU/HA—or PLGA scaffolds seeded with MSCs. In another similar study using PU NPs Lin et al. have developed thermoresponsive 3D bioink of waterborne PCL based biodegradable PU with a soft segment replaced with 20 mol% of PLLA diol or poly(D,L-lactide) (PDLLA) diol to form two different PU NPs (Lin et al., 2016). To decrease gelation time soy protein isolate (SPI) was also blended with PU NPs. Neural stem cells (NSCs) were embedded within the ink prior to printing. The cell laden bioprinted NSCs in the PU/SPI hybrid hydrogel have shown better cell viability and proliferation than those in the PU gel. Same group has also investigated the effect of graphene on the PU hydrogels for neural tissue engineering (Huang et al., 2017). It has been reported that addition of graphene nanomaterials at very low amount (25 ppm) into the hydrogel considerably improved the oxygen metabolism (2- to 4-fold increase) along with the neural differentiation of neural stem cells in 3D printed constructs. A similar study based on dual (photo/thermo) responsive PU NPs has reported that hydrogels were developed with tunable mechanical properties upon heating the biodegradable PU NPs (Hsiao and Hsu, 2018). Cells can be easily added to the bioink prior to printing for production of cell-laden scaffolds using microextrusion-based 3D printing due to shear thinning characteristic of hydrogels at 37°C. A handable (tofu-like) and stable scaffold with inductive 3D microenvironment for differentiation of neural stem cells can be obtained using softer hydrogels with low modulus (< 1 kPa).

**Figure 9:**
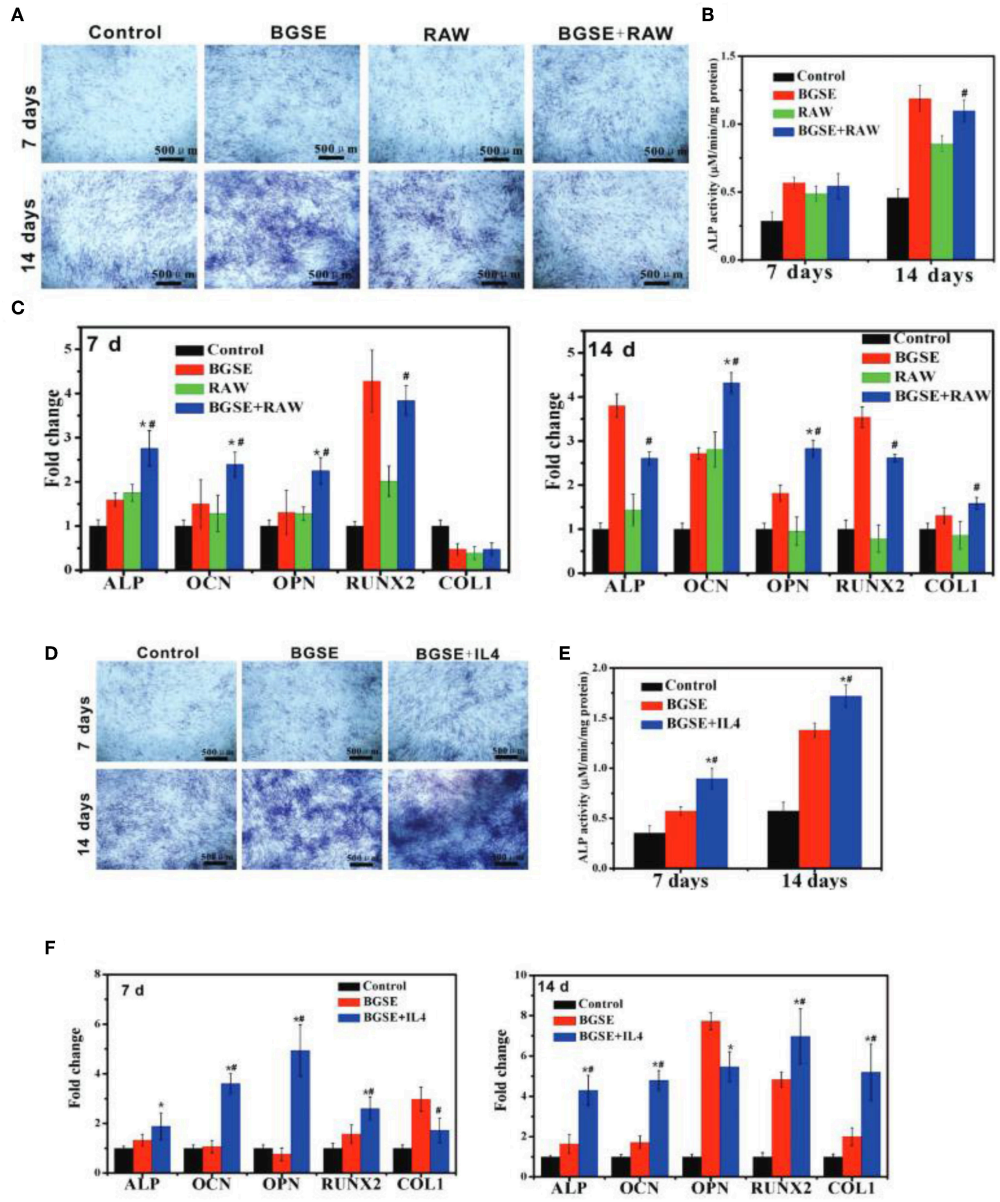
**(A-C)** Osteogenic differentiation of 3T3 cells under inflammatory state. **(A)** ALP staining and **(B)** ALP quantitative assay of 3T3 cells after cultured for 7 and 14 days. **(C)** mRNA expression of osteogenesis-related genes (ALP, OCN, OPN, COL1, RUNX2) in 3T3 cells cultured for 7 and 14 days. ^*^p < 0.05 vs. BGSE (bioactive glass scaffolds extracts) group; ^#^*p* < 0.05 vs. RAW (macrophage-like cell line) group. **(D–F)** Osteogenic differentiation of 3T3 cells in anti-inflammatory state. **(D)** ALP staining and **(E)** ALP quantitative assay of 3T3 cells after being treated with various samples for 7 and 14 days. **(F)** mRNA expression of osteogenesis-related genes (ALP, OCN, OPN, COL1, RUNX2) of 3T3 cells incubated with various groups for 7 and 14 days. ^*^p < 0.05 vs. blank control; ^#^*p* < 0.05 vs. BGSE group. Reproduced from Zhao et al. (2018) with permission from John Wiley and Sons.

Application of 3D printing for bone tissue applications generally requires, heating or chemical procedures during fabrication due to mechanical constraints which limits the encapsulation of cells in scaffolds. To rectify these restrictions bioink printed hydrogel scaffolds have been developed for bone grafts. These scaffolds can mimic the bone extracellular matrix for cell migration, proliferation and differentiation but lack enough mechanical strength for bone tissue application arising from nature of biomaterials used in bioink. Incorporation of nBGs and HAp NPs can increase compressive moduli of the scaffolds. Gao et al. have produced a poly(ethylene glycol) dimethacrylate (PEGDMA) 3D printed scaffold containing bioactive glass (BG) 45S5 or HAp NPs for bone marrow derived human MSCs encapsulation (Gao et al., 2014). Scaffolds containing HAp NPs have shown higher, compressive modulus, cell viability, production of collagen and ALP activity by the cells in comparison to BG 45S5 group after 21 days of culturing. In a recent study Zhao et al. have synthesized nBGs and 3D printed by mixing nBGs with PVA and investigated the effect of this structure on immunomodulation associated mechanism of osteogenesis in the extramembranous which occurs outside the cortical bone (Zhao et al., 2018). Peripheral macrophage-conditioned medium used in culturing calvaria preosteoblasts following the stimulation with nBGs. It promoted migration of preosteoblast, but osteogenic differentiation was effected restrictedly. On the other hand, the anti-inflammatory milieu and nBGs considerably increased preosteoblasts' osteogenic differentiation (Figures 9A–F). *In vivo* results have shown active extramembranous osteogenesis.

## Conclusion

*In vivo* maturation of engineered tissues requires a well synchronized series of events involving host immune system, circulatory system and cellular component of the implanted material. Physicochemical properties of the scaffolds and the presence/immobilization of bioactive agents within the scaffolds can help in achieving a precise control over maturation. Herein, we reviewed the use of nanoscale structures with different material properties for enhancing the properties of tissue engineering scaffolds by rendering them antimicrobial, providing sequential release of several growth factors, by concentrating necessary contrasting agents for monitoring, by improving mechanical properties of bioprinted structures as bioink supplements and also by providing structural elements such a HAp in nanoscale to induce tissue specific reactions. The future tissue engineering solutions might contain multiple components for all these aspects to have higher control over integration, monitoring and long-term safety of engineered tissues. Focus of nanoscale delivery systems should not only be on manufacturing advanced delivery systems but also on also on evaluating systemic cytotoxic effect and immune responses of these systems. Such studies could provide better understanding of biocompatibility of vast numbers of nanoscale delivery systems and will direct the future studies with higher success rate in cost-effective approach. For this aim, the different nanoparticle types of different materials (metals, ceramics, magnetic/paramagnetic materials and polymers) provide a strong toolbox for tissue engineered artificial organ functionalization.

## Author Contributions

NV proposed the topic. MF-A, HK-M, CRdS, and JB wrote the initial and revised drafts. EB, AT, and NV provided feedback through the writing process and completed revisions based on feedback provided.

### Conflict of Interest Statement

The authors declare that the research was conducted in the absence of any commercial or financial relationships that could be construed as a potential conflict of interest.
